# Effect of Penetration Enhancers on Drug Nail Permeability from Cyclodextrin/Poloxamer-Soluble Polypseudorotaxane-Based Nail Lacquers

**DOI:** 10.3390/pharmaceutics10040273

**Published:** 2018-12-13

**Authors:** Elena Cutrín-Gómez, Soledad Anguiano-Igea, M. Begoña Delgado-Charro, José Luis Gómez-Amoza, Francisco J. Otero-Espinar

**Affiliations:** 1 Department of Pharmacology, Pharmacy and Pharmaceutical Technology, University of Santiago de Compostela, Santiago de Compostela 15782, Spain; elenacutrin@hotmail.com (E.C.-G.); s.anguiano.igea@gmail.com (S.A.-I.); joseluis.gomez.amoza@usc.es (J.L.G.-A.); 2 Department of Pharmacy and Pharmacology, University of Bath, Bath BA2 7AY, UK; B.Delgado-Charro@bath.ac.uk (M.B.D.-C.)

**Keywords:** transungual drug delivery, nail, medicated nail lacquers, onychomycosis, nail psoriasis, polypseudorotaxanes, methyl-β-cyclodextrin, poloxamers, ciclopirox olamine, clobetasol propionate, penetration enhancer

## Abstract

Nail delivery has interest for local treatment of nail diseases. Nevertheless, the low permeability of drugs in the nail plaque precludes the efficacy of local treatments. The use of penetration enhancers can increase drug permeability and improve the efficacy of the treatment of nail pathologies. In this work, different chemical substances have been evaluated as potential penetration enhancers. With this aim, the effect of different substances such as sodium lauryl sulfate (SLS), polyethylene glycol 300 (PEG 300), carbocysteine, *N*-acetylcysteine, lactic acid, potassium phosphate, Labrasol® and Labrafil® in the microstructure, nail surface and drug permeability has been evaluated. The models obtained by mercury intrusion porosimetry and PoreXpert™ software show a more porous structure in nails treated with different enhancers. Permeation studies with bovine hooves and nails revealed that all the hydroalcoholic lacquers developed, and particularly those prepared with SLS, provide better nail penetration of the drugs ciclopirox olamine and clobetasol propionate. Results have shown that the increase of the drug penetration in the nail is caused by the formation of a porous random microstructure and by the decrease of the contact angle between lacquers and the surface or the nail plaque. The presence of SLS produces an improvement in the spreading of the solution on the nail surface and promotes the penetration of the solution into the nail pores. The hydroalcoholic lacquer, elaborated with cyclodextrin/poloxamer soluble polypseudorotaxane and sodium lauryl sulfate as an enhancer, allowed the rate of diffusion and penetration of the active ingredient within the nail to be significantly higher than obtained with the reference lacquers when using either ciclopirox olamine or clobetasol propionate as the active ingredient.

## 1. Introduction

Ungual drug delivery has been receiving increasing attention in the past few years [1,2]. This is caused by the need to have topical formulations available that improve drug penetration in the nail structure and, consequently, the efficacy of treatment of nail pathologies.

The objective pursued with an ungual topical therapy is the attainment of, at least, the minimum therapeutic concentration in every layer of the nail relevant to the pathology considered, including the deepest ones. This is a challenging task since the nail layer, besides being much thicker than the stratum corneum, presents completely different physicochemical and structural characteristics [3]. For this reason, dermal topical formulations present very low or even no efficacy in ungual drug delivery since the methods used to improve drug penetration into the skin are ineffective in this area of administration [4,5,6,7].

The nail plate is mainly composed of keratinocytes with different degrees of keratinization depending on the location. The superficial (dorsal) area of the plate consists of dead cells that have lost their nucleus and store a great amount of fibrous proteins, such as keratin, which provides elasticity and resistance. The intermediate area consists of cells that are strongly bound to one another by desmosomes and hemidesmosomes and contains high proportions of keratinized cells, although the cells contain a smaller amount of keratin than those of the superficial area. Lastly, the lower (ventral) area, which rests on the nail bed, consists of two layers of cells stemming from the epidermic bed [8,9,10,11]. Finally, nails have a lower proportion of intercellular lipids than skin [12]. For these reasons, the nail plate represents a more hydrophilic barrier than the skin [12].

Incorporation of certain chemicals into ungual topical formulations might modify the nail plate structure, promoting drug penetration and increasing the efficacy of topical therapies. It is not surprising, therefore, that chemical methods have been explored together with physical and mechanical enhancement approaches to promote drug penetration across the nail plate [1,3,13]. Because mechanical methods, including abrasion and avulsion of the nail, have poor patient acceptance since they are traumatic, painful and invasive, most recent research has focused on finding new non-invasive physical methods and chemical procedures [14,15].

Chemical methods involve incorporating permeation enhancers that increase drug penetration into the nail into the formulation [16]. A priori, permeation enhancers, normally used in dermal and transdermal delivery, are expected to be ineffective on the nail for the reasons above. Thus, the search for chemicals that facilitate drug ungual penetration, the main objective of this study, is especially timely [2,3].

So far, few substances with the capacity of promoting penetration into the nail have been described. For example, *N*-acetylcysteine [17,18], phosphoric acid [19] or fungal keratinase [20] were proven to promote drug penetration through bovine hooves, this being a nail model typically used for preliminary studies [21]. Likewise, molecular screenings have been carried out in order to identify possible candidates for ungual enhancers [19,22,23,24]. Table 1 summarizes the state of the art and collects the most frequent enhancers described in the literature as well as a brief description of their mechanism of action.

The aim of this work is to identify better penetration enhancers to promote the drug nail absorption from heat-sensitive water–alcohol hydrogels based on soluble polypseudorotaxanes of Poloxamer 407 and soluble derivatives of the β-cyclodextrin. We have previously shown that these vehicles form a hydrated film coating on the surface of the nail, facilitating the hydration of the nail plate and the release and diffusion of the drug into the nail [25,26,27].

Based on these preliminary studies, our starting point was the selection of compounds with, in principle, greater potential as ungual penetration enhancers (lactic acid, sodium lauryl sulfate, PEG 300, cysteine, carbocysteine and potassium phosphate), along with other chemicals considered to be efficacious enhancers of transdermal and oral routes of administration such as Labrasol® [28] and Labrafil® [29]. The candidates have been the subject of several studies (mercury intrusion porosimetry and scanning electron microscopy) that aimed at identifying and characterizing the possible alterations that they produce on the microstructure and composition of nail components (RAMAN spectroscopy). The efficacy of these enhancers in enabling drug penetration into the nail was evaluated through diffusion studies involving bovine hoof membranes and human nail clippings. Two model drugs were selected: clobetasol propionate, a drug of choice in the treatment of nail psoriasis [30], and ciclopirox olamine, used for the treatment of onychomycosis [31].

## 2. Materials and Methods 

### 2.1. Materials

The enhancers tested were sodium lauryl sulfate (Fagron Iberica, Tarrasa, Spain), polyethylene glycol 300 (Merck Millipore, Darmstadt, Germany), Labrasol® and Labrafil® (Gattefossé SAS, Madrid, Spain), lactic acid and sodium phosphate (VWR, BDH Prolabo, Barcelona, Spain), carbocysteine and acetylcysteine (Acorfarma, Madrid, Spain). The drugs used were Ciclopirox olamine (Fagron Iberica, Barcelona, Spain), clobetasol-17-propionate (Acorfarma, Spain). The compounds used in the elaboration of the vehicles were Poloxamer 407 (Pluronic® F127, Sigma-Aldrich-Merck, Darmstadt Germany), partially methylated β-cyclodextrin (Crysmeb®, Roquette Laisa, Valencia, Spain), ethanol (Merck Millipore) and purified water (Helix® Millipore).

The phosphate-buffered saline was prepared, following the 9th edition of the *European Pharmacopoeia*, from potassium dihydrogen phosphate, sodium chloride, and sodium dihydrogen phosphate dodecahydrate, all of them of analytical quality. Sodium azide was added to the buffer to prevent microbial growth in permeation studies; this was from Panreac Quimica SA (Barcelona, Spain). Finally, methanol from Prolabo (VWR, BDH Prolabo, Barcelona, Spain) was used to extract ciclopirox olamine and clobetasol-17- propionate from nails and hooves.

Bovine hooves were obtained from the local slaughterhouse (Compostelana de Carnes S.L; Santiago de Compostela, Spain). The hooves were cleaned with water and kept frozen. Before the experiments, they were defrosted and hydrated in order to facilitate the process of cutting them (Ufesa Professional Slicer FS50, Ufesa, Barcelona, Spain) into small slices (0.3–0.7 mm thick), which were dried at room temperature.

Nail samples were obtained from volunteers between the ages of 25 and 65. The volunteers signed an informed consent form for the use of the samples, and their use was authorized by the Ethics Committee of Galicia (Project identification code: 2018/099; date of approval: 22 February 2018). The volunteers cut their own fingernails and toenails and provided them to the researchers. The samples were meticulously cleaned and washed with water, dried at room temperature and stored in a glass container also at room temperature. The nail samples used in diffusion studies were approximately 8 mm long, whereas the samples used in the rest of the studies were between 1 and 3 mm.

### 2.2. Nail Incubation in the Presence of the Enhancers

The nail samples were immersed for 24 h at room temperature, in aqueous solutions containing 5% of the enhancers. The one exception was carbocyisteine, which, due to its low solubility, was tested at 0.2% (Table 2). Following the incubation, the nails were cleaned with water and freeze-dried in liquid nitrogen (Telstar LyoQuest Plus, Telstar, Terrassa, Spain).

The nail samples (both treated and non-treated) were subjected to the following tests:

### 2.3. Scanning Electron Microscopy (SEM)

The samples were placed on adhesive graphite and copper disks and were analyzed using a Zeiss Evo LS 15 SEM microscope. Images from both the dorsal and ventral surface were taken from randomly chosen locations of the surface.

The analysis of the microphotographs from the surface of the samples was carried out using Image Pro Plus 6 software (Media Cybernetics Inc., Rockville, MD, USA) from 10 SEM images of the nail surface.

### 2.4. Mercury Intrusion Porosimetry (MIP)

The samples were analyzed in a Micromeritics Autopore IV porosimeter (Norcross, GA, USA) equipped with a penetrometer with 3-mL capacity, and an analysis pressure interval of 0.004 to 172.4 MPa was used. The amount of combined nails used in each study was, approximately 0.6 g, which may amount to 50–60 specimens depending on the size. The porosimeter gives the pore size distribution data and the % of porosity of samples. The data regarding pore size distribution were modeled using PoreXpert™ software (Environmental and Fluid Modelling Group, University of Plymouth, UK). A detailed description of the methods can be found elsewhere [17].

The permeability to water and the rate of penetration of water into the nail were estimated based on the modeled structures, as previously described [17]. To calculate the permeability, enhancer solutions were used to first determine the surface contact angle between the solutions and the nail to include this value in the PoreXpert simulation.

To identify the layers in PoreXpert nail models that can act as a limiting step in molecular diffusion, each model structure was divided into 20 parallel horizontal planes. Each layer occupies one-tenth of the thickness of the entire model. Thus, the surface occupied by the pores, connections and bottlenecks in each of the 10 layers of the models was calculated from the PoreXpert data. Finally, the percentage of the surface of each layer occupied by the pores, connections and bottlenecks relative to the total layer surface was determined. This value is the critical surface value.

### 2.5. RAMAN Spectroscopy

Three sets of spectra from each nail surface were obtained using a Raman Bruker Fourier transform Scope spectroscope. The spectra were taken from three randomly chosen locations on the ventral and dorsal surfaces of the nail. The ratio between the height of the signal, corresponding to the C–C (900 cm^−1^) and S–S (500 cm^−1^) vibrations, was calculated.

### 2.6. Determining the Contact Angle through Goniometry

Solutions of the enhancers at the same concentrations used in the incubation and diffusion studies (Table 2) have been tested in an SEO Phoenix 300 (Surface Elector Optics, Gyeonggi-do, Korea). For this, a small drop of solution has been placed on the surface of nail samples and then a series of photographs have been taken, so the evolution of the drop over 10 s can be observed. The contact angle with the nail, for each of the enhancers, has been determined by the analysis of the obtained images. The angle value has been selected based on the moment at which the drop becomes stable on the nail surface.

### 2.7. Permeation Studies

The aqueous lacquers used in the in vitro diffusion studies were prepared according to previous studies [27]. A 1:1 water–ethanol solution was prepared containing 10% *w*/*v* of partially methylated β-cyclodextrin and 5% *w*/*v* of poloxamer 407. Next, the enhancer (Table 2) was incorporated and stirred until it was completely dissolved. Lastly, the drug, either ciclopirox olamine or clobetasol-17-propionate, was added in excess and the vehicles were continuously stirred for 24 h. Finally, the formulations were filtered through nylon membranes of 0.45 µm (Merck Millipore) to eliminate the undissolved drug. The concentration of ciclopirox olamine in the formulations prepared is determined through UV spectroscopy (Hewlett-Packard 8452A, Hewlett-Packard Española, S.A, Madrid, Spain) and the concentration of clobetasol propionate through Ultra Performance Liquid Chromatography –mass spectrometry UPLC-LC/MS (see below).

In a first step to assess the efficacy of the enhancers, a model of bovine hooves was used [39,40]. In a second phase, the enhancers showing higher efficacy were tested using human nail samples. Bovine hooves and human nails permeation studies were performed with vertical Franz penetration cells (Vidrafoc, Barcelona, Spain) with ~7 mL receptor solution. For this, prior to the test, hoof slices and human samples were hydrated in water for two hours to provide them with flexibility so they could be clamped between two Teflon cylindrical adapters (polytetra-fluoroethylene, Mecanizados del Noroeste, Santiago de Compostela, Spain), providing an effective diffusional area of 0.196 cm^2^. Then, adapters with samples were clamped between the donor and receptor chambers of Franz diffusion cells. The thickness of the slices was measured with a micrometer (Mitutuyo, Guipúzcoa, Spain). Samples with a similar thickness and without visible faults or fissures were selected for the studies. The average thickness was 0.4–0.7 for nails and 0.4–0.8 mm for hooves.

The solutions containing the enhancers (2 mL each) were added to the donor compartment. The receiving solution for ciclopirox olamine was phosphate-buffered saline (pH 7.4; 37 ± 0.5 °C), to which sodium azide (30 mg/L) had been added to prevent algae and microbiological growth. Sink conditions were maintained in all the tests as the drug concentration in the receiver was always less than 10% of the drug’s PBS solubility. In the case of clobetasol propionate, the receiving solution was PBS containing 5% HPB with sodium azide (30 mg/L) to increase drug solubility in the receptor.

The diffusion studies lasted 11 days; 1-mL samples were obtained from the receiver every 24 h, and the same volume was replaced with fresh PBS. The receptor samples were filtered (0.45 µm nylon filter) before analysis. In the case of ciclopirox olamine, samples from tests with human nails were diluted with NaOH 1 M to prevent interferences with protein residues coming from the nail extraction.

Once the diffusion studies were finished, the amount of ciclopirox olamine or clobetasol propionate present inside the nail or hoof was determined. In order to do this, and after dismantling the diffusion cells, the samples were cleaned with distilled water and then dried with cellulose paper. Then, the sections of nails or hooves exposed to the lacquer were cut into small pieces, which were weighed and incorporated into 10-mL vials, to which 5 mL phosphate buffer with methanol at 5% were added. Then, the vials were incubated at a temperature of 25 °C and under continuous stirring for six days to facilitate the drug extraction.

The concentration of ciclopirox olamine in the samples was spectrophotometrically quantified at 308 nm (Diode-Array Spectrophotometer Hewlett Packard 8452 (Hewlett-Packard Española, S.A, Madrid, Spain). Regarding the clobetasol propionate, quantification was performed on a MS/MS tandem Waters Xevo® TQD detector linked to an Acquity UPLC® H-Class system (Waters®, Czech Republic) using TargetLynxTM Application Manager. An Acquity BEH C18 column (2.1 × 50 mm, 1.7 µm particle size, Waters® Czech Republic) at 40 °C was used, using isocratic conditions: water–methanol 20:80 at 0.5 mL/min. The autosampler was kept at 10 °C and the volume of injection was 5 µL. Acquisition of mass spectrometric data was in multiple reaction monitoring mode (MRM) via positive electrospray ionization. Ion transitions of m/z 467.1 > 355.1 (Cone voltage 100 V, collision energy 10 V) a desolvation gas flow of 1100 l/h, cone gas of 80 l/h and capillary voltage of 0.55 kV were used for acquisition. The desolvation and source temperature were 450 °C and 146 °C, respectively. The amounts of accumulated drug diffused (M) to the receiver according to the type (t) were normalized in accordance with the diffusion area (A) (0.196 cm^2^).

## 3. Results and Discussion

**Figure 1:**
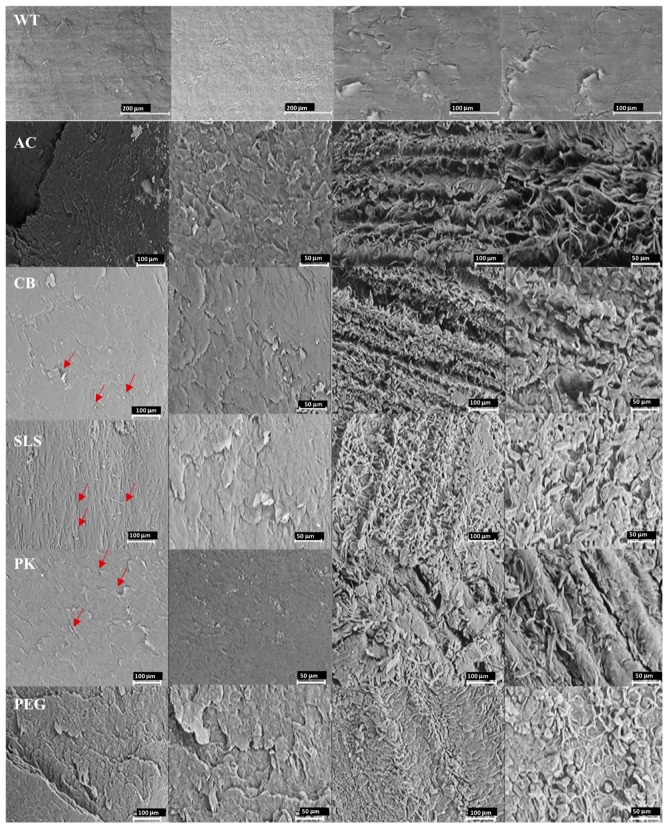
Scanning electron microscopy photomicrographs at different magnifications of the healthy nail plates incubated with the enhancers (WT: nail without treatment, AC: acetylcysteine, CB: carbocysteine, SLS: sodium lauryl sulfate, PK: potassium phosphate, and PEG: polyethylene glycol 300) on their external (dorsal) surface (two first left columns) and on the internal (ventral) surface (two right columns). Red arrows point to some of the pores.

Figure 1 shows SEM images of the dorsal and ventral surfaces of healthy nails treated with the different enhancers studied.

Photomicrographs were taken at different augmentations in order to accurately determine the size of the pores and identify fractures or alterations on the surface. Healthy nails appear to be compact and present a surface with small pores and fissures.

Superficial porosity were estimated based on these SEM photomicrographs (Table 3). In all cases, the appearance of the surface and the size of the surface pores were similar to each other.

**Figure 2:**
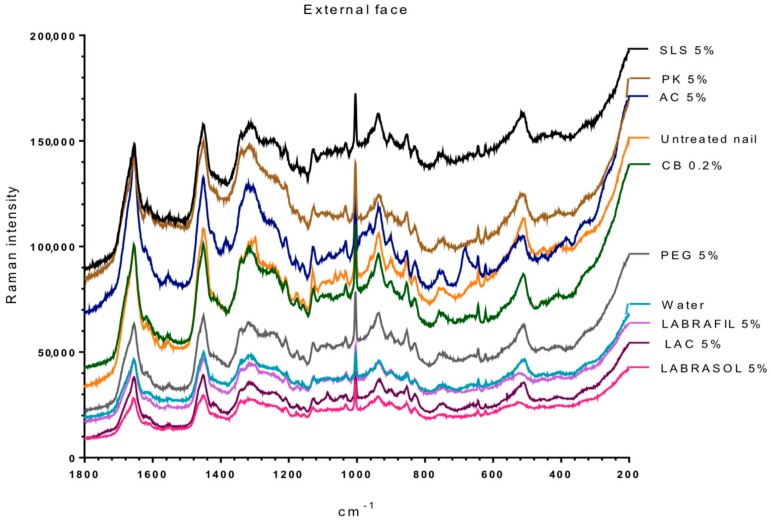
RAMAN spectra of the external (dorsal) surface of nails treated with the different enhancers in the range of 200–1800 cm^−1^. AC: acetylcysteine, CB: carbocysteine, SLS: sodium lauryl sulfate, PK: potassium phosphate, PEG: polyethylene glycol 300, LAC: lactic acid.

**Figure 3:**
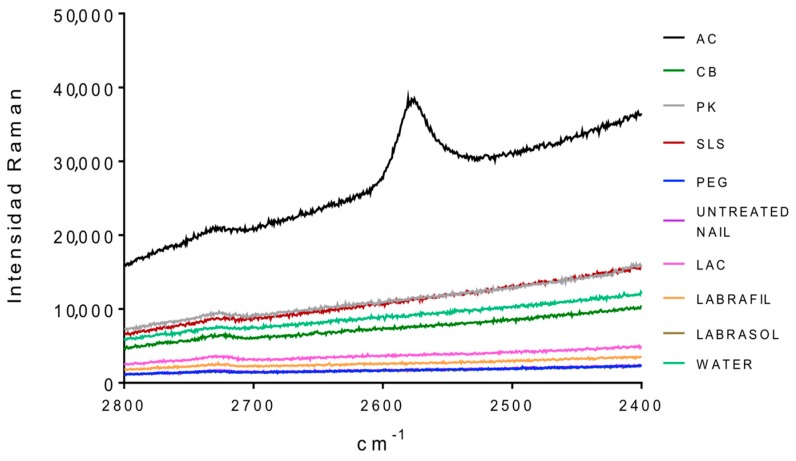
RAMAN spectra in a 2400–2800 cm^−1^ interval of the external nail surface treated with the different enhancers. AC: acetylcysteine, CB: carbocysteine, SLS: sodium lauryl sulfate, PK: potassium phosphate, PEG: polyethylene glycol 300, LAC: lactic acid.

Figure 2 and Figure 3 show the Raman spectra of the surface of nails treated with the different enhancers. All samples showed the characteristic spectrum of protein structures, similar to the one obtained by Wessel et al. [41] and by ourselves in previous studies [26,27] for the healthy nail and after being hydrated with water. The spectra were typical of the keratins or main components of the nail plate, that is, the vibrations corresponding to C–C bonds at a wave number slightly above 900 cm^−1^, to hydroxyl groups between 1600 and 1700 cm^−1^, to disulfide bridges S–S around 500 cm^−1^ and to amide or amine groups at 1300 and 1600 cm^−1^.

Table 3 shows the ratio value between the height of the band signal, corresponding to the vibration frequency of the S–S bonds and of the C–C bonds (υ_ss_/υ_cc_) for the dorsal and ventral surfaces of the nails untreated and treated with different enhancers. No significant differences were observed between treatments for the ratio values (one way Analysis of the Variance, α n.s.) suggesting no significant alteration in the S–S bonds. However, the analysis of the bands corresponding to the group –SH (2600 cm^−1^, Figure 3) shows that a significant occurrence of these groups happened when the nail was treated with *N*-acetylcysteine. The occurrence of the –SH is related to disulfide bridges. As a consequence, the acetylcysteine is the only compound affecting these bridges, whereas the formation of of -SH radicals was not observed in the rest of the enhancers.

**Figure 4:**
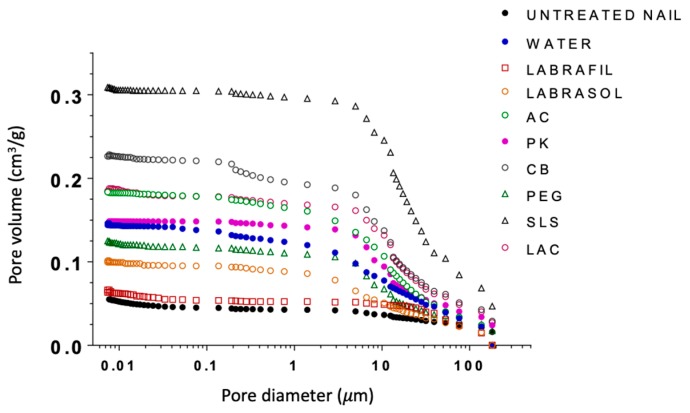
Cumulative curves of porous volume obtained through mercury intrusion porosimetry for healthy nails treated with the different enhancers. AC: acetylcysteine, CB: carbocysteine, SLS: sodium lauryl sulfate, PK: potassium phosphate, PEG: polyethylene glycol 300, LAC: lactic acid.

Mercury intrusion porosimetry was used to investigate potential modifications occurring in the microporous structure of the nails following the enhancers’ treatment. Mercury intrusion porosimetry tests were performed with both treated and untreated nails. To use this technique, it is essential to completely eliminate the water from the pores while keeping the microstructure of the nail intact and allowing the intrusion of the mercury. Therefore, after the 24-h treatment the nails were rapidly frozen by immersion in liquid nitrogen and then freeze-dried. Pore size distributions corresponding to the nails treated with the enhancers are shown in Figure 4 and the total porosity in Table 4.

With the exception of Labrafil, Labrasol and PEG, the porosity of untreated nails and hydrated nails was much lower than that measured for nails treated with enhancers. Likewise, clear differences were observed between the enhancers. The porosity of nails was ranked as: treated with SLS > CB > LAC~AC > PK > water.

The cumulative curves for PEG and water-treated nails were very similar. Lastly, nails treated with Labrasol® had higher porosity than those treated with Labrafil®; both groups exhibited lower porosities than hydrated nails. Labrafil® and untreated nails showed similar porosity profiles.

**Figure 5:**
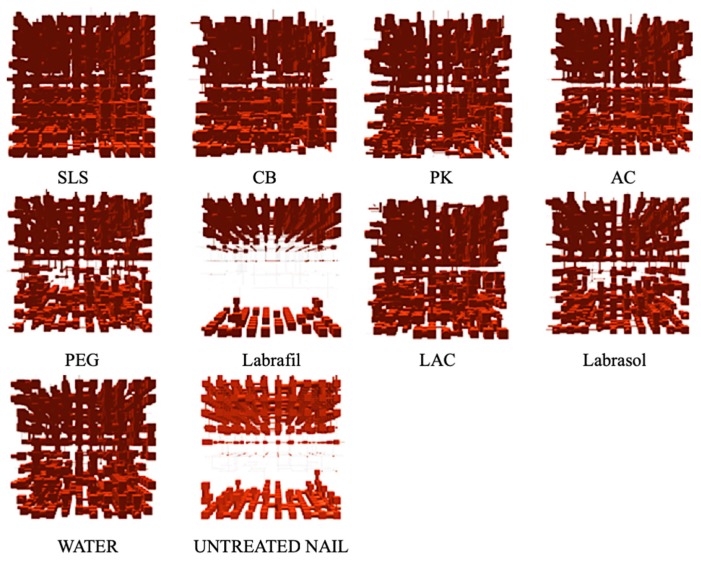
Models for ungual structures for nails treated with different enhancers and for untreated controls obtained through PoreXpert™. AC: acetylcysteine, CB: carbocysteine, SLS: sodium lauryl sulfate, PK: potassium phosphate, PEG: polyethylene glycol 300, LAC: lactic acid.

Porous structures were modeled based on the cumulative curves of mercury intrusion porosimetry using PoreXpert™ software. Figure 5 shows the single cell structures resulting from this modeling, in which the pores are represented by cubic spaces connected through cylindrical bottlenecks (this being the same representation as in the mercury intrusion analysis).

In agreement with our previous findings [17,27] models of untreated nails showed a very compact internal area, with very few and very small pores, throats and bottlenecks that inhibit drug penetration. The model corresponding to nails incubated with Labrafil® showed a very similar internal structure, indicating that this excipient had little effect on the microstructure of the nails.

Also consistent with previous findings [17,27], water incubation caused swelling of the nail plate, resulting in a significant increase in the number and size of the pores of the superficial layers as well as of the internal layers of the models. This structure, illustrated by the model in Figure 5, will facilitate molecular diffusion. Similar model structures were obtained with PEG or, to a lesser extent, with Labrasol®. Lastly, the structural models corresponding to nails treated with enhancers such as PK, AC, CB, LAC and especially SLS, showed an increase in pore number, size and number of connections, thus suggesting that those are the most effective enhancers.

The correlation value obtained with PoreXpert® for the three-dimensional models is a working parameter related to the level of spatial order of the pores in the material. This parameter ranges from 0, when the spatial order of the structure is completely random, to 1, when the single cell is perfectly ordered. The correlation value for the model structure for healthy nails was 0.692 (Table 4), indicating a significant level of order in the structure, in accordance with the occurrence of perfectly defined areas with very different porosities (porous in the external and internal surfaces and barely porous in internal areas).

Slightly higher values (0.816 and 7.54% porosity) were obtained for the model corresponding to nails treated with Labrafil®, which is expected given the small structural differences observed and suggests inhibition of the water diffusion and nail hydration. Additional experiments are necessary to elucidate the mechanism by which Labrafil® prevents the nail hydration. Nail hydration induced the formation of a more porous, disordered and random structure; thus, the correlation value decreased to 0.159 and the porosity increased to 15.24%, a similar value to that of nails treated with PEG 300. Lastly, the inclusion of enhancers such as SLS, AC, LAC or PK reduces the correlation value to values below 0.1, increasing the % porosity in the majority of cases.

**Figure 6:**
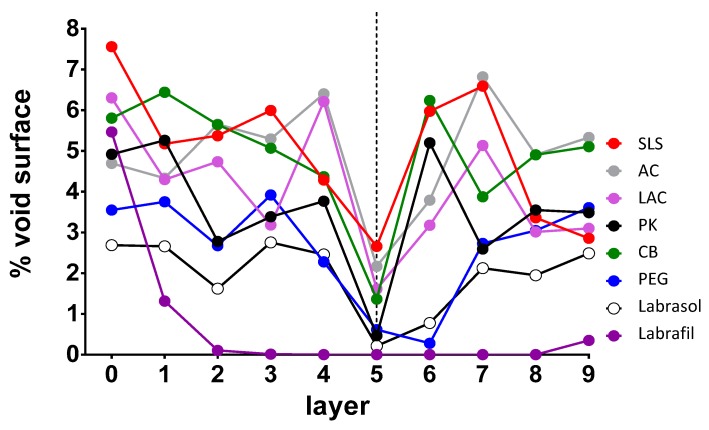
Representation of the critical surface obtained for each enhancer depending on the assessed plane. 0 represents the upper surface of the model and 9 the lower surface. AC: acetylcysteine, CB: carbocysteine, SLS: sodium lauryl sulfate, PK: potassium phosphate, PEG: polyethylene glycol 300, LAC: lactic acid. The dashed line represents the critical surface layer that has the minimum values of the void surface in the majority of model samples.

The formation of this type of random microstructure facilitates drug penetration by fostering the disappearance of the less porous internal zone of the nail models that acts as a diffusional barrier. An attempt was made to identify the layers of the nail model that can act as a limiting step in molecular diffusion. The critical surface of the connections and bottlenecks was determined for each of them, with the results shown in Figure 6. For all the enhancers, relatively high values of critical surface were observed on the most superficial layer that represents the nail’s surface, with the highest value observed for SLS. As we move towards the internal part of the nail modeled structures, a decrease in the surface values was observed, especially for Labrafil®, in which case barely any bottleneck surface was observed from the third layer. This decrease would suggest a greater resistance to water and other molecules’ diffusion through the structure. For most enhancers, the decrease was observed from the intermediate layer 5. However, for AC and SLS, the minimum values of critical surface found are still high, so a lower resistance to the diffusion of water molecules or of active ingredients is expected compared with Labrasol®, Labrafil®, PK or PEG. LAC and CB have intermediate behavior.

**Figure 7:**
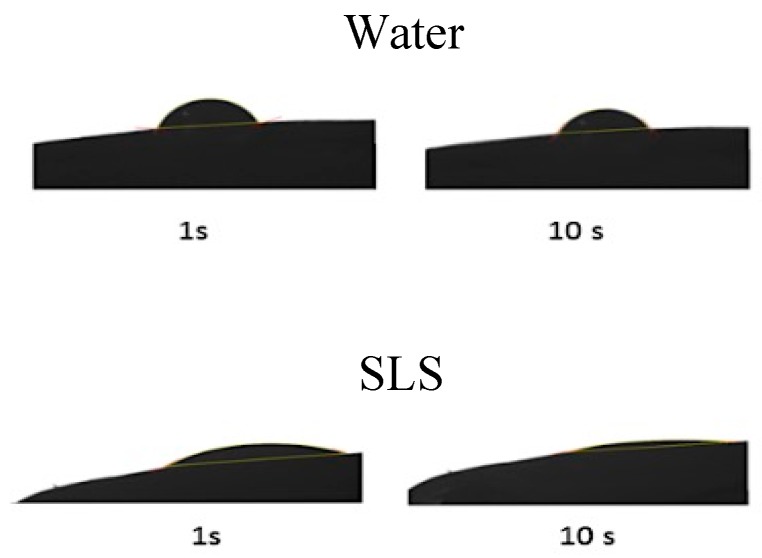
Example of images of the angle obtained at different times with water and SLS.

Water diffusion into the modeled-nail structures was simulated, taking the porosity model obtained through MIP and PoreXpert™ software into consideration. Prior to doing this, it was necessary to determine the contact angle between the nail plate and the solutions with the enhancers (Figure 7 and Table 5) as a measure of the surface tension between the two. This parameter was used by PoreXpert to make the diffusion simulation, using them as a measure of the spreading capacity of the solutions over the nail, which is related to the penetration rate of the water into the pores and channels of the material. For most of the enhancers, a contact angle similar to that of water was obtained, with values ranging from 65° to −70°. However, this value was considerably lower for the SLS solution; the greater spreading capacity of this surfactant solution could facilitate the entrance of water and chemicals into the ungual structure.

**Figure 8:**
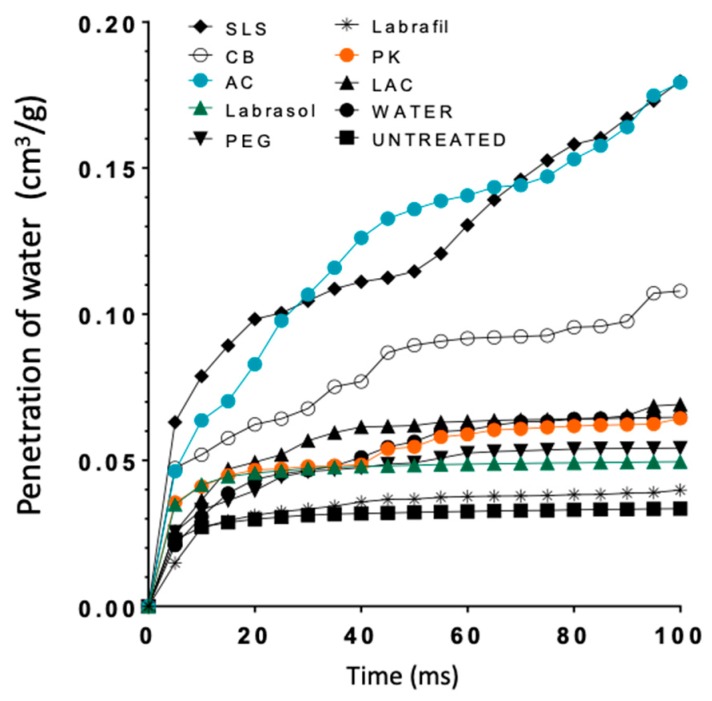
Curves representing water penetration through the nail as a function of time obtained from the PoreXpert™ models. AC: acetylcysteine, CB: carbocysteine, SLS: sodium lauryl sulfate, PK: potassium phosphate, PEG: polyethylene glycol 300, LAC: lactic acid.

Figure 8 shows the curves simulating water penetration through the modeled-nail structures. An increased penetration rate was observed for the enhancers SLS and AC, and an intermediate behavior for CB; similar profiles were predicted for the rest of the enhancers and the hydrated nail. Finally, Labrafil® was an exception; the profile observed for this excipient was identical to that simulated for untreated nails. SLS and AC, and to a lesser extent CB, are the promoters that have a greater value of % void surface in all the layers of the model and those that have greater porosity values in the critical surface layer (Figure 6), which facilitates the penetration of water into the nail structure.

The results obtained so far suggested that both SLS and AC were the excipients with the highest potential as enhancers. However, others such as LAC, CB or PEG could potentially yield good results. In order to verify these predictions, diffusion studies on bovine hoof models were performed. To simulate a practical application, the absorption enhancers were included into the base of a hydroalcoholic lacquer previously developed and characterized [27], and the maximum loading capacity of the drug ciclopirox olamine into the lacquers was determined.

Table 6 shows the thermodynamic apparent solubility of ciclopirox olamine in the lacquers. The highest loading values were obtained for vehicles with PK 5%, SLS, AC 5%, PEG 5% and CB 0.2%, suggesting that they have, to some extent, a solubilizing effect on the ciclopirox olamine compared with LAC, Labrasol® and Labrafil®. For comparison purposes, the table shows the content of ciclopirox olamine for two marketed products, as provided in their label information (SmPC).

The two marketed products were used as a reference, and the 10 CPO-saturated lacquers with enhancers were used for permeation experiments on bovine hooves and human nails. The cumulative permeation profiles for CPO across the bovine hoof (see Figure S1) indicate that the new lacquer provided superior permeation of the drug compared to the reference formulation (Ony-Tec®). Two-way ANOVA and Tukey multiple comparison tests on the amounts of diffused ciclopirox olamine showed the following significant differences (α < 0.05): (a) between the SLS 5% lacquer and the reference formulation after seven days, (b) between each of the new SLS 5%, AC 5%, CB 0.2% and PEG 300 5% lacquers and the reference formulation after 11 days. The flux of the ciclopirox olamine across the hoof (Table 6) was calculated from the slope of the cumulative permeated drug versus time profiles (see Figure S1) in the 1–7-day time period test (where profiles are linear). A lacquer containing the tested enhancers yields faster penetration profiles and higher diffusion coefficients than Ony-Tec, the reference formulation. The use of SLS 5% produces the highest values of all the enhancers.

The values of % of dose delivered from the lacquers through the hoof and the nail are similar to those obtained with cyclodextrin/poloxamer polyseudorrotaxanes aqueous lacquers containing 5% AC obtained in previous studies [25] (2.4% of the dose). Nevertheless, the presence of ethanol and some enhancers increased the loading concentration from 7.7 mg/mL to values higher than 20 mg/mL, so that the drug released in the nail is significantly higher. Also, the drug flux with respect to the previously reported value [25,27] (57.6 and 48 or 16.72 µg cm^−2^ day^−1^ in hoof or nail, respectively) is higher.

**Figure 9:**
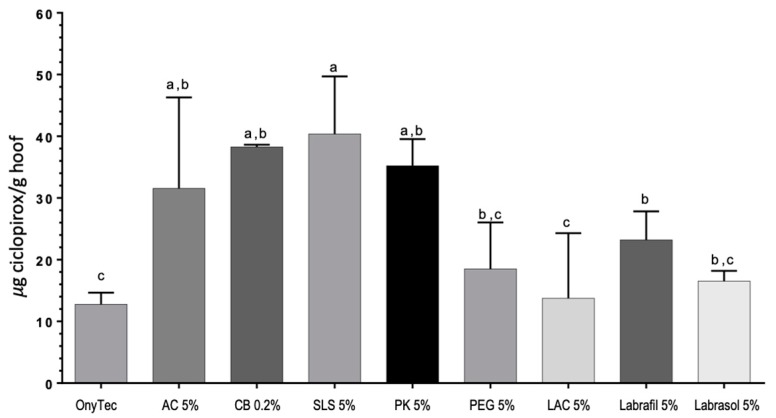
Amount of ciclopirox olamine (µg CPO/g of hoof) recovered from the bovine hoof slices at the end of the diffusion experiments. The same letters (a, b or c) indicate homogeneous groups ANOVA, Tukey post hoc test, α < 0.05). AC: acetylcysteine, CB: carbocysteine, SLS: sodium lauryl sulfate, PK: potassium phosphate, PEG: polyethylene glycol 300, LAC: lactic acid.

Moreover, Figure 9 shows increased levels of ciclopirox olamine in the interior of the hoof after the 11-day study for the SLS 5%, CB 0.2% and PK 5% lacquers as compared to the reference Onytec and the LAC 5% lacquer.

The results with hoof slices identified SLS as the enhancer with the highest potential in the group tested. However, the lacquers containing 5% of SLS looked cloudy and showed yellowing a few hours after preparation. Previous work [42,43] has described how SLS can interact with cyclodextrins to form inclusion complexes; thus, SLS might compete with the drugs for the cavity causing drug precipitation.

To prevent precipitation, the concentration of SLS in the lacquers was reduced and, accordingly, the effect of SLS on the drug diffusion was tested over the range 0.5–1.0% as it led to completely transparent lacquers.

**Figure 10:**
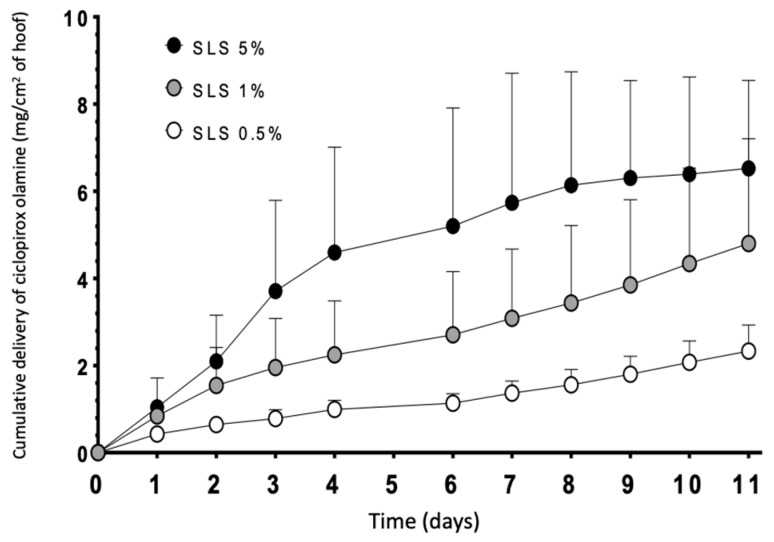
Diffusion profiles of ciclopirox olamine through the bovine hoof, using lacquers with different SLS concentrations. Data correspond to mean +/− SD (*n* = 3).

Figure 10 shows the results of the diffusion tests performed with lacquers with different concentrations of SLS. Diffusion of ciclopirox olamine through the hoof increased with the SLS content in the lacquer; significant differences in the cumulative amount of drug recovered in the receptor from the six days was found (two-way ANOVA, α < 0.05) between the lacquers incorporating 0.5% and 5% of SLS. No other significant differences were found. Thus, for this reason, higher permeation of the drug was obtained with the 5% lacquer containing SLS; the intermediate concentration of 1% was selected to perform the studies on human nails because precipitation was not observed.

**Figure 11:**
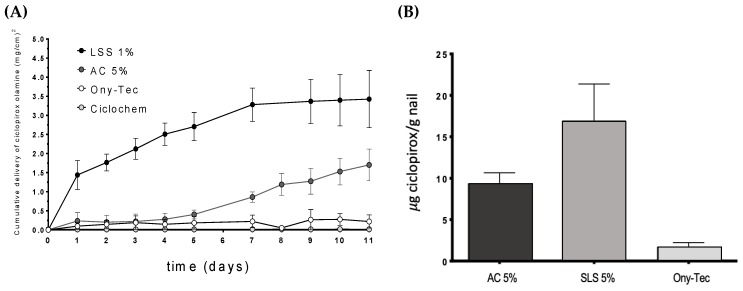
Diffusion profiles of ciclopirox olamine in the nail (**A**) and amount (µg/g of nail) retained in the nail plate after 11 days (**B**). SLS: sodium lauryl sulfate, AC: acetylcysteine.

The permeation profiles of ciclopirox olamine through the nail (Figure 11) showed the superiority of the hydroalcoholic lacquers containing SLS compared to two marketed products Ony-Tec and Ciclochem used as a reference and also compared to the lacquer with the enhancer AC. Significant differences (α < 0.05) on the amount of drug recovered in the receptor were found from the first day of the study and over the 11 days. The lacquers with AC show a similar profile to that of Ony-Tec over the first days, but subsequently provided a higher flux from the sixth day of the test, so the amount of diffused drug by the end of the study is significantly bigger (α < 0.05). The order of the drug flux values in the nail (Table 6) were SLS > AC > Ony-Tec > Ciclochem. SLS lacquer shows similar flux in nails and hooves.

Similarly, the amount of ciclopirox olamine recovered from the nails at the end of permeation tests was highest for the SLS lacquer, and lowest for the reference product Ony-Tec. In the case of Ciclochem, it was impossible to precisely determine the amount within the nail because of the difficulties in eliminating the polymer layer from the nail surface without causing artifacts.

**Figure 12:**
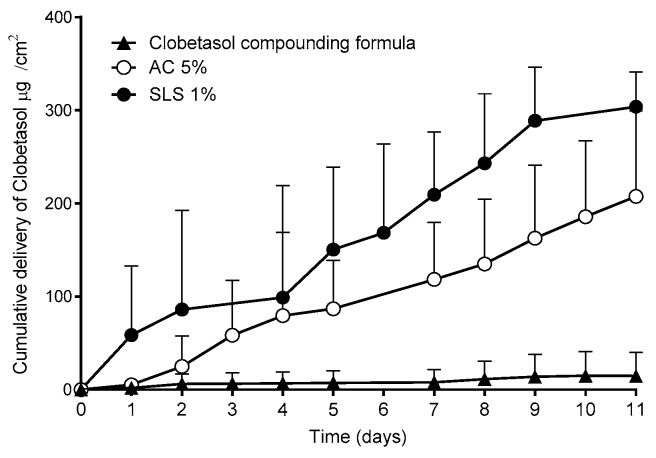
Permeation profiles of clobetasol propionate and the amount accumulated in the nail after 11 days of study. SLS: sodium lauryl sulfate, AC: acetylcysteine.

A further study investigated whether the superior performance on the new lacquers described above could be applicable to other drugs. Thus, lacquers containing clobetasol propionate, a corticosteroid used in the treatment of ungual psoriasis, were prepared. Since no approved products containing this drug are available, a compounding formula dispensed to treat this pathology was used [44]. This compounding formula is prepared by mixing the drug with a marketed lacquer base. The results of the permeation studies performed with the nails are shown in Figure 12.

Consistent with the results obtained with CPO, the lacquers with SLS provided significantly higher (α < 0.05) clobetasol propionate permeation fluxes into the nail than those of the reference compounding formula.

**Figure 13:**
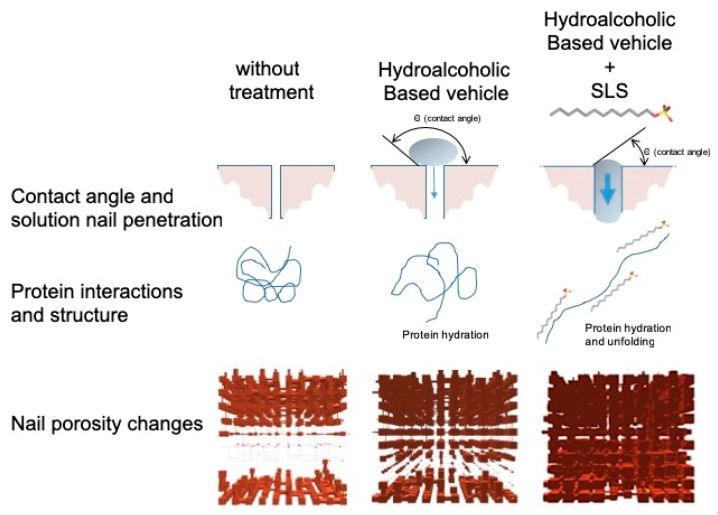
The suggested mechanism for how hydroalcoholic vehicles containing SLS can enhance drug penetration into the nail.

The use of sodium lauryl sulfate as an enhancer has yielded excellent results, showing great potential to improve the efficiency of the formulations for drug transungual delivery. The improvement observed when using this surfactant may be due to the modifications produced in the secondary structure of the keratins, modifying the microporous structure of the nail plaque, and to the decrease in the contact angle between the lacquer and the nail, which improves spreading and lacquer penetration into the nail pores, promoting the diffusion of the active ingredient (Figure 13).

## 4. Conclusions

The chemicals LAC, PK, AC, CB and SLS significantly modified the structure of the nails and hooves, making them more permeable to drug diffusion. This capability identifies them as penetration enhancers for the nail.

The models obtained by mercury intrusion porosimetry and PoreXpert™ show a more porous structure in the nails treated with enhancers than for those chemicals without enhancement effects and control untreated nails. Raman spectroscopy studies suggested that, with the exception of *N*-acetylcysteine, the modifications of the microporosity could not be attributed to the rupture of disulfide bridges and that other mechanisms were involved in the increase in porosity. Permeation studies with bovine hooves and nails revealed that all the hydroalcoholic lacquers developed, and particularly those prepared with SLS, have superior performance as drug delivery systems compared to the reference lacquers used in current therapies.

The hydroalcoholic lacquer elaborated with a sodium lauryl sulfate content of 1% allowed the rates of diffusion and penetration of the active ingredient within the nail to be significantly higher than those obtained with the reference lacquers when using either ciclopirox olamine or clobetasol propionate as the active ingredient.

## 5. Patents

This work is part of the submitted patent WO2015185647A1.

## Figures and Tables

**Table 1:** Common nail penetration enhancers described in the bibliography.

Enhancer	Proposed Mechanism of Action	Reference
Sodium lauryl sulfate (SLS)	Denaturation of proteins because of electrostatic interactions or micelle formation	[32]
Polyethylene Glycol 300 (PEG)	Increase in the hydration and swelling of the nail	[33]
Carbocysteine (CB)	Reduction of keratin’s disulfide bridges	[34]
*N*-acetylcysteine (AC)	Reduction of keratin’s disulfide bridges	[34,35]
Lactic Acid (LAC)	Formation of micropores	[19,34]
Potassium Phosphate (PK)	Increases hydration of the nail and the thermodynamic activity of the drug	[33]
Labrasol®	Enhancer of absorption for transdermal release	[36,37]
Labrafil®	Enhancer of absorption for transdermal release	[38]

**Table 2:** Chemicals and concentrations tested as enhancers of nail penetration. SLS was tested at 5% in incubation experiments and at three levels in diffusion experiments.

Enhancer	Concentration (% weight/volume)
Sodium lauryl sulfate (SLS)	5/1/0.5
PEG 300 (PEG)	5
Carbocysteine (CB)	0.2
*N*-acetylcysteine (AC)	5
Lactic Acid (LAC)	5
Potassium Phosphate (PK)	5
Labrasol®	5
Labrafil®	5

**Table 3:** Values of the ratio value between the height of the band signal corresponding to the vibration frequency of the S–S bonds and of the C–C bonds (υ_ss_/υ_cc_) for external (dorsal) and internal (ventral) surfaces of the nail exposed to the enhancers’ solutions for a 24-h period.

Enhancer	Surface Pore Size	υ_ss_/υ_cc_	
	Geometric Mean (Geometric Standard Deviation)	Dorsal	Ventral
Untreated nail	4.20 (0.41)	1.07	1.04
Water	3.91 (0.39)	1.01	1.05
SLS 5%	4.34 (0.37)	0.87	0.94
PEG 300 5%	4.01 (0.40)	0.97	0.86
CB 0.2%	3.69 (0.37)	0.95	0.98
AC 5%	3.87 (0.37)	0.96	1.02
LAC 5%	0.37 (0.39)	1.04	1.02
PK 5%	4.06 (0.33)	0.98	1.03
Labrasol® 5%	-	0.93	0.83
Labrafil® 5%	-	0.90	0.83

**Table 4:** Porosity and correlation values for untreated and treated nails.

Enhancer	Porosity (%)	Correlation
Untreated nail	6.98	0.692
Hydrated nail	15.24	0.159
SLS 5%	28.20	0.074
PEG 300 5%	13.90	0.194
CB 0.2%	22.75	0.124
AC 5%	27.86	0.053
LAC 5%	19.14	0.052
PK 5%	16.14	0.012
Labrasol® 5%	11.27	0.200
Labrafil® 5%	7.54	0.816

**Table 5:** Values of the contact angle (mean +/− SD, *n* = 3) between the nail and the aqueous solutions of the different enhancers.

Absorption Enhancer	Average Contact Angle (°)
Water	69.15 ± 10.59
AC	66.60 ± 9.33
LAC	62.55 ± 9.23
CB	66.08 ± 7.38
PK	66.51 ± 8.06
SLS	30.71 ± 8.05
PEG	65.31 ± 6.03
Labrafil®	56.43 ± 34.12
Labrasol®	50.84 ± 14.29

**Table 6:** Loading values of the lacquers (mg/mL) and results of diffusion studies conducted with ciclopirox olamine through nails and hooves. The content from OnyTEC and Ciclochem was taken from the product information.

Enhancer	Ciclopirox Olamine Concentration(mg/mL)	Hoof		Nail	
		Flux mg/day·cm^2^	% Dose Delivered	Flux mg/day·cm^2^	% Dose Delivered
AC 5%	24.31 ± 3.45	0.734	1.95	0.164	0.68
CB 0.2 %	22.50 ± 1.56	0.605	2.68		
PEG 5%	23.81 ± 3.25	0.582	2.33		
PK 5%	36.85 ± 1.54	0.409	0.70		
SLS 0.5%	21.17 ± 2.19	0.181	0.86		
SLS 1 %	23.47 ± 3.92	0.561	2.00	0.308	1.36
SLS 5%	26.45 ± 2.21	1.325	3.02		
LABRAFIL 5%	17.89 ± 2.16	0.477	2.64		
LABRASOL 5%	16.18 ± 3.19	0.507	3.14		
LAC 5%	14.85 ± 2.19	0.555	2.68		
Ony-Tec	80	0.158	0.21	0.011	0.09
Ciclochem	80			0.000	0.006

## Supplementary Materials

The following are available online at http://www.mdpi.com/1999-4923/10/4/273/s1, Figure S1. Diffusion profiles of ciclopirox olamine through the bovine hoof, using lacquers with different penetration enhancers. Data correspond to mean +/− SD (*n* = 3).
