# Supplementary figures and images for: Effect of Penetration Enhancers on Drug Nail Permeability from Cyclodextrin/Poloxamer-Soluble Polypseudorotaxane-Based Nail Lacquers
Source: Pharmaceutics. 2018 Dec 13;10(4):273. doi: 10.3390/pharmaceutics10040273 (PMC6321522; doi:10.3390/pharmaceutics10040273)

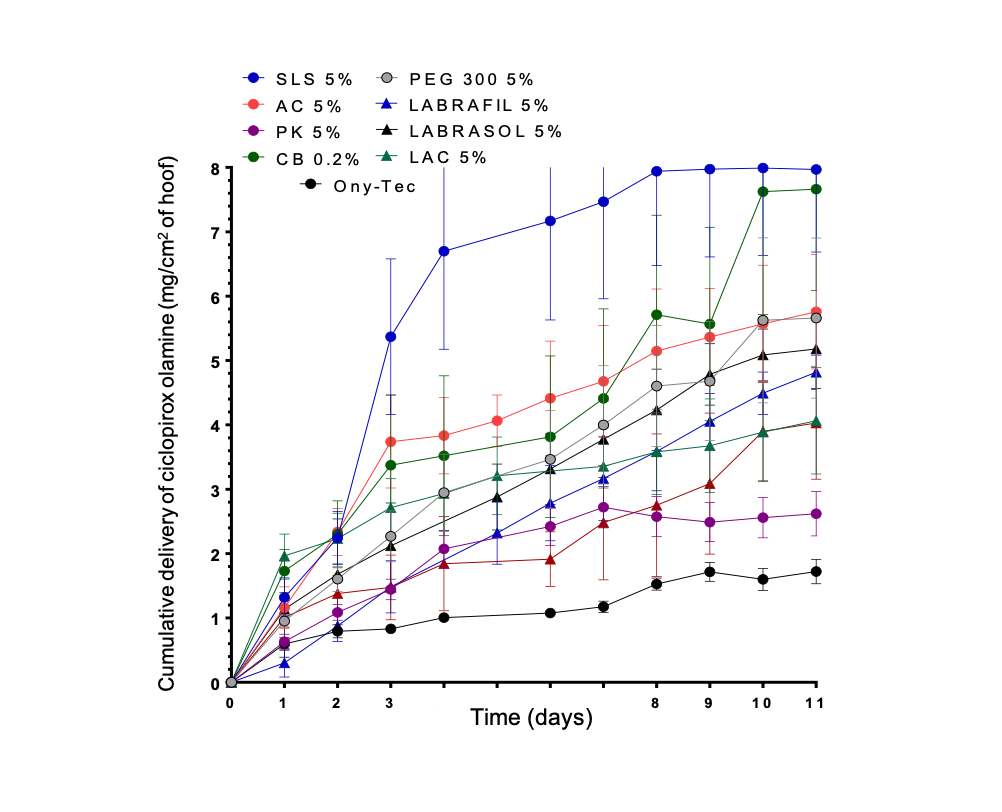

Supplement: Supplementary file 1 [file pharmaceutics-10-00273-s001.zip › Figure S1.tif]
